# Association of *PPARG* rs 1801282 C>G polymorphism with risk of colorectal cancer: from a case-control study to a meta-analysis

**DOI:** 10.18632/oncotarget.20138

**Published:** 2017-08-10

**Authors:** Jiakai Jiang, Zhiqiang Xie, JunYing Guo, Yafeng Wang, Chao Liu, Sheng Zhang, Weifeng Tang, Yu Chen

**Affiliations:** ^1^ Department of General Surgery, Changzhou No. 3 People’s Hospital, Changzhou, Jiangsu Province, China; ^2^ Department of Clinical Laboratory, Fujian Medical University Union Hospital, Fuzhou, Fujian Province, China; ^3^ Department of Clinical Laboratory, Fujian Cancer Hospital, Fujian Medical University Cancer Hospital, Fuzhou, Fujian Province, China; ^4^ Department of Cardiology, The People's Hospital of Xishuangbanna Dai Autonomous Prefecture, Jinghong, Yunnan Province, China; ^5^ Department of Cardiothoracic Surgery, Affiliated People’s Hospital of Jiangsu University, Zhenjiang, Jiangsu Province, China; ^6^ Cancer Bio-immunotherapy Center, Fujian Cancer Hospital and Fujian Medical University Cancer Hospital, Fuzhou, Fujian Province, China; ^7^ Department of Medical Oncology, Fujian Cancer Hospital and Fujian Medical University Cancer Hospital, Fuzhou, Fujian Province, China; ^8^ Fujian Provincial Key Laboratory of Translational Cancer Medicine, Fuzhou, Fujian Province, China

**Keywords:** PPARG, polymorphism, colorectal cancer, risk

## Abstract

The functional single nucleotide polymorphisms in *peroxisome proliferator-activated receptor gamma* (*PPARG*) gene were predicted to be correlated with the susceptibility of colorectal cancer (CRC). The aim of the present study was to explore the relationship between *PPARG* rs1801282 C>G polymorphism and the risk of CRC. First, we conducted a case-control study with 387 CRC cases and 1,536 controls. We used the SNPscan method to determine the genotypes of *PPARG* rs1801282 C>G polymorphism. We found *PPARG* rs1801282 C>G polymorphism had a tendency of decreased risk to CRC risk (CG vs. CC: adjusted OR, 0.67, 95% CI = 0.43–1.04 for CG vs. CC, *P* = 0.073; GG vs. CC: adjusted OR, 0.68; 95% CI, 0.44–1.05; *P* = 0.078). The stratified analysis revealed *PPARG* rs1801282 C>G polymorphism also had a tendency of decreased risk to colon cancer (CG *vs*. CC: adjusted OR = 0.54, 95% CI = 0.27–1.08, *P* = 0.083). The results of subsequent meta-analysis suggested that *PPARG* rs1801282 C>G polymorphism might be a protective factor for CRC, especially in Asians, colon cancer and rectum cancer subgroups. In conclusion, our study indicates that *PPARG* rs1801282 C>G polymorphism might decrease the risk of overall CRC. Larger sample size and well-designed case-control studies are needed to confirm the potential association.

## INTRODUCTION

Colorectal cancer (CRC) is the fifth most common type of malignancy among males and the fourth most common type among females in China, accounting for 215,700 and 160,600 cases in 2015, respectively [1]. The incidence of CRC is rapidly increasing in developing countries including China [1, 2]; however, the etiology of CRC remians unknown. Risk factors, such as family history of CRC, advanced age, inflammatory bowel diseases, benign adenomatous polyps, being physically inactive, drinking, smoking, high intake of dietary fat and low intake of vegetables and fruits, may play important roles in the development of CRC [3–9]. Accumulating evidence suggested that besides individual lifestyle and environmental factors, some genetic factors may be relevant to the etiology of CRC.

The gene of peroxisome proliferator-activated receptor gamma (*PPARG*), a ligand-activated transcription factor, is located in 3p25. PPARG shares conservative domain with other steroid receptors (e.g., the vitamin D, estrogen, progesterone, retinoid and thyroid receptors), which recognize to peroxisome proliferator-activated receptor (PPAR) response elements in the region of promoter, and then bind to them. Subsequently, these steroid receptors regulate the transcription of some target genes. It is well known that PPARG may be involved in controlling adipocytes differentiation, regulating energy homeostasis, influence of cellular cholesterol homoeostasis, and the development of type 2 diabetes mellitus (T2DM) and obesity [10–12].

Many investigations evidenced the potential roles of *PPARG* gene in determining CRC susceptibility. Understanding the variants in this gene correlated with CRC susceptibility may be helpful for CRC prevention and diagnosis. Recently, some case-control studies focused on the relationship of *PPARG* polymorphisms with the risk of CRC. A common single nucleotide polymorphism in *PPARG* gene [rs1801282 C>G (Pro12Ala)] have been established, which were associated with receptor activity, insulin sensitivity, body mass index (BMI), and risk of T2DM [13, 14]. Many studies focused on the association of *PPARG* rs1801282 C>G polymorphism with risk of CRC. Several meta-analyses demonstrated that *PPARG* rs1801282 C>G polymorphism was associated with the decreased risk of CRC in Caucasians [15, 16]. However, there were only three case-control studies with relatively small sample sizes focused on the relationship between *PPARG* rs1801282 C>G polymorphism and CRC in Asians [17–19]. The evidence may be limited.

The biological significance of PPARG indicates that functional polymorphisms in *PPARG* gene may influence the susceptibility of CRC. Thus, the attempt of the present study was to assess the relationship of rs1801282 variations in *PPARG* with CRC risk. The results of our case-control study might be limited by sample size. With the aim to overcome this limitation, a comprehensive pooled-analysis was subseqently carried out to determine the association of *PPARG* rs1801282 C>G polymorphism with CRC risk.

## RESULTS

### Study characteristics

Table 1 summarized the distribution of demographic variables and risk factors in CRC cases and controls. We found there was no significant difference in the distributions of age (cases: 60.21 ± 12.48, vs. controls: 60.82 ± 8.82; *P* = 0.272), sex (*P* = 0.213), smoking (*P* = 0.505) and alcohol consumption (*P* = 0.058) between cases and controls. CRC patients have relatively lower body mass index (BMI) than that of the control subjects (*P* < 0.001). When it comes to TMN stages, according to AJCC criteria from 2010, 196 and 191 CRC patients were classified as stage I/II and III/IV, respectively. The primary information of *PPARG* rs1801282 C>G polymorphism was listed in Table 2. The genotype distributions of *PPARG* rs1801282 C>G polymorphism in controls were in accordance with HWE (*P* = 0.544).

**Table 1:** Distribution of selected demographic variables and risk factors in colorectal cancer cases and controls

Variable	Cases (*n* = 387)		Controls (*n* = 1,536)		*P*^a^
	*n*	%	*n*	%	
Age (years)	60.21 (± 12.48)		60.82 (± 8.85)		0.272
Age (years)					0.502
< 61	186	48.06	709	46.16	
≥ 61	201	51.94	827	53.84	
Sex					0.213
Male	236	60.98	989	64.39	
Female	151	39.02	547	35.61	
Smoking status					0.505
Never	270	69.77	1098	71.48	
Ever	117	30.23	438	28.52	
Alcohol use					0.058
Never	335	78.55	1381	89.91	
Ever	52	21.45	155	10.09	
BMI (kg/m^2^)	22.70 (± 3.16)		24.05 (± 3.15)		< 0.001
BMI (kg/m^2^)					< 0.001
< 24	263	67.96	775	50.46	
≥ 24	124	32.04	761	49.54	
Site of tumor					
Colon cancer	169	43.67			
Rectum cancer	218	56.33			
Degree of differentiation^b^					
Low	56	16.28			
Medium	261	75.87			
High	27	7.85			
Lymph node status					
Positive	177	45.74			
Negative	210	54.26			
TMN stage					
I + II	196	50.65			
III + IV	191	49.35			

^a^Two-sided χ^2^ test and student *t* test; BMI, body mass index; FPG, fasting plasma glucose; HDL-C, high-density lipoprotein cholesterol; LDL-C, low-density lipoprotein cholesterol; ^b^six subjects have missing data.

**Table 2:** Primary information of the *PPARG* rs1801282 C>G polymorphism

Genotyped SNPs	*PPARG* rs1801282 C>G
Chromosome	3
Chr Pos (NCBI Build 37)	12393125
Function	missense
MAF for Chinese in database	0.07
MAF in our controls (*n* = 1,536)	0.05
*P* value for HWE^f^ test in our controls	0.544
Genotyping method	SNPscan
% Genotyping value	99.64

MAF: minor allele frequency;

HWE: Hardy–Weinberg equilibrium.

### Association of *PPARG* rs1801282 C>G polymorphism with CRC risk

Table 3 summarizes the genotype distributions of *PPARG* rs1801282 C>G polymorphism in CRC cases and controls. The genotype frequencies of *PPARG* rs1801282 C>G were 93.21% (CC), 6.53% (CG) and 0.26% (GG) in CRC patients, which were not significantly different from those in non-cancer controls (90.28% CC, 9.39% CG and 0.33% GG). When compared with the frequency of *PPARG* rs1801282 CC genotype, individuals carrying the CG genotype had a tendency of decreased risk to CRC risk (crude OR = 0.67, 95% CI = 0.43–1.04 for CG vs. CC, *P* = 0.072). When compared with the frequency of *PPARG* rs1801282 CC genotype, individuals carrying the GG genotype also had this tendency to CRC risk (crude OR = 0.68, 95% CI = 0.44–1.04 for GG vs. CC, *P* = 0.077). Adjustments for age, sex, smoking, drinking and BMI, the observed tendency was not essentially changed (CG vs. CC: adjusted OR, 0.67, 95% CI = 0.43–1.04 for CG vs. CC, *P* = 0.073; GG vs. CC: adjusted OR, 0.68; 95% CI, 0.44–1.05; *P* = 0.078; Table 4). Results of other genetic comparisons are listed in Table 4.

**Table 3:** The frequencies of *PPARG* rs1801282 C>G polymorphism in colorectal cancer patients and controls

Genotype	CRC cases (*n* = 387)		Colon cancer (*n* = 169)		Rectum cancer (*n* = 218)		Controls (*n* = 1,536)	
	*n*	%	*n*	%	*n*	%	*n*	%
CC	357	93.21	157	94.01	200	92.59	1,384	90.28
GC	25	6.53	9	5.39	16	7.41	144	9.39
GG	1	0.26	1	0.60	0	0	5	0.33
GC+GG	26	6.79	10	5.99	16	7.41	149	9.72
CC+GC	382	99.74	166	99.40	216	100.00	1,528	99.67
GG	1	0.26	1	0.60	0	0	5	0.33
G allele	27	3.52	11	3.29	16	3.70	154	5.02

**Table 4:** Overall and stratified analyses of *PPARG* rs1801282 C>G polymorphism with colorectal cancer by region

Genotype	Overall colorectal cancer cases (*n* = 387) vs. controls (1,536)				Colon cancer (*n* = 169) vs. controls (1,536)				Rectum cancer (*n* = 218) vs. controls (1,536)			
	Crude OR (95% CI)	*P*	Adjusted OR^a^ (95%CI)	*P*	Crude OR (95% CI)	*P*	Adjusted OR^a^ (95% CI)	*P*	Crude OR (95% CI)	*P*	Adjusted OR^a^ (95% CI)	*P*
Additive model	0.67 (0.43–1.04)	0.072	0.67 (0.43–1.04)	0.073	0.55 (0.27–1.09)	0.086	0.54 (0.27–1.08)	0.083	0.76 (0.45–1.31)	0.324	0.77 (0.45–1.32)	0.335
Homozygote model	0.77 (0.09–6.60)	0.810	0.77 (0.09–6.82)	0.814	1.75 (0.20–15.03)	0.612	1.80 (0.21–15.73)	0.596	–	–	–	–
Dominant model	0.68 (0.44–1.04)	0.077	0.68 (0.44–1.05)	0.078	0.59 (0.31–1.15)	0.120	0.59 (0.30–1.14)	0.117	0.76 (0.45–1.31)	0.324	0.77 (0.45–1.32)	0.335
Recessive model	0.80 (0.09–6.87)	0.839	0.80 (0.09–7.07)	0.838	1.84 (0.21–12.85)	0.579	1.89 (0.22–16.57)	0.566	–	–	–	–

^a^Adjusted for age, sex, smoking status, alcohol use and BMI status in a logistic regression.

### Association of *PPARG* rs1801282 C>G polymorphism with CRC risk in a stratification group by site of tumor

To assess the effect of *PPARG* rs1801282 C>G polymorphism in different tumor site, a stratified analysis was conducted. The stratified analysis revealed *PPARG* rs1801282 C>G polymorphism also had a tendency of decreased risk to colon cancer (CG *vs.* CC: adjusted OR = 0.54, 95% CI = 0.27–1.08, *P* = 0.083; Table 4).

### Meta-analysis of *PPARG* rs1801282 C>G polymorphism and CRC risk

**Figure 1:**
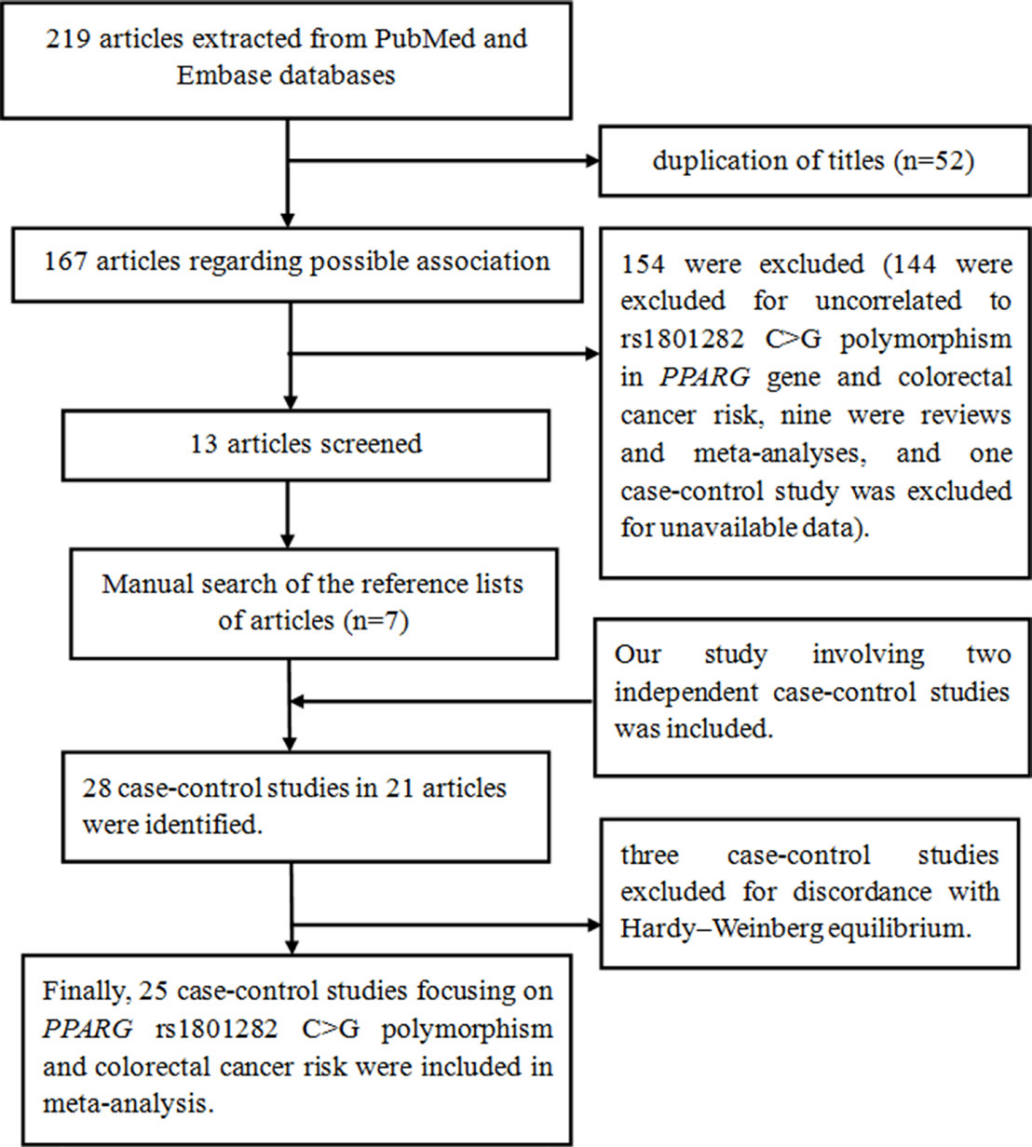
Flow chart of study selection procedure

Next, a comprehensive meta-analysis was carried out to determine the relationship between *PPARG* polymorphisms and CRC risk. In total, 219 abstracts were retrieved from Pubmed and EMBASE databases. The detailed selecting process is summarized in Figure 1. There were several subgroups in our present study and some publications [17, 19–22], we treated them separately. The detailed characteristics and *PPARG* rs1801282 genotypes of included studies are listed in Table 5. Finally, our present study and previously published studies involving 12,761 cases and 21,113 controls were recruited in this pooled-analysis.

**Table 5:** Characteristics of the eligible studies included in the meta-analysis for *PPARG* rs1801282 C>G polymorphism

Study	Year	Country	Ethnicity	Type	Case/Control	Case			Control			HWE
						CC	CG	GG	CC	CG	GG	
Our study	2016	China	Asians	Colon cancer	169/1,536	157	9	1	1,384	144	5	Yes
Our study	2016	China	Asians	Rectum cancer	218/1,536	200	16	0	1,384	144	5	Yes
Crous-Bou et al.	2012	Israel	Caucasians	Colorectal cancer	1,780/1,864	710	102	0	1,307	163	9	Yes
Sainz et al.	2012	German	Caucasians	Colorectal cancer	1,801/1,783	1,354	415	32	1,334	427	22	Yes
Abuli et al.	2011	Spain	Caucasians	Colorectal cancer	515/502	426	87	2	419	80	3	Yes
Tsilidis et al.	2009	USA	Caucasians	Colorectal cancer	208/381	165	37	1	295	68	6	Yes
Kury et al.	2008	France	Caucasians	Colorectal cancer	1,023/1,121	822	194	7	896	212	13	Yes
Vogel et al.	2007	Denmark	Caucasians	Colorectal cancer	355/753	252	96	7	550	190	13	Yes
Kuriki et al.	2006	Japan	Asians	Colorectal cancer	128/238	120	7	0	221	17	0	Yes
Theodoropoulos et al.	2006	Greece	Caucasians	Colorectal cancer	222/200	164	48	10	118	70	12	Yes
Slattery et al.	2006	USA	Caucasians	Colorectal cancer	2,371/2,972	1,840	496	35	2,283	645	44	Yes
Siezen et al.	2006	Netherlands	Caucasians	Colorectal cancer	204/399	160	40	1	325	70	2	Yes
Siezen et al.	2006	Netherlands	Caucasians	Colorectal cancer	487/750	387	92	8	596	146	8	Yes
Gunter et al.	2006	USA	Caucasians	Colorectal cancer	244/231	151	54	4	141	52	3	Yes
Koh et al.	2006	Singapore	Asians	Colon cancer	206/1,164	195	11^*^	-	1,057	89^*^	-	Yes
Koh et al.	2006	Singapore	Asians	Rectum cancer	156/1,164	150	6^*^	-	1,057	89^*^	-	Yes
McGreavey et al.	2005	UK	Caucasians	Colorectal cancer	478/733	366	80	9	403	100	10	Yes
Jiang et al.	2005	India	Asians	Colon cancer	59/291	46	13	0	230	57	4	Yes
Jiang et al.	2005	India	Asians	Recum cancer	242/291	194	44	4	230	57	4	Yes
Gong et al.	2005	USA	Caucasians	Colorectal cancer	163/212	129	30	4	153	52	7	Yes
Murtaugh et al.	2005	USA	Caucasians	Colon cancer	1,577/1,971	1,234	343^*^	-	1,493	478^*^	-	Yes
Murtaugh et al.	2005	USA	Caucasians	Recum cancer	794/1,001	606	188^*^	-	790	211^*^	-	Yes
Landi et al.	2003	Spain	Caucasians	Recum cancer	139/326	111	15	3	243	61	5	Yes
Landi et al.	2003	Spain	Caucasians	Colon cancer	238/326	200	31	0	243	61	5	Yes
Smith et al.	2001	UK	Caucasians	Recum cancer	37/49	37	3	0	49	11	2	Yes

^*^GG+CG;

HWE: Hardy-Weinberg equilibrium.

**Figure 2:**
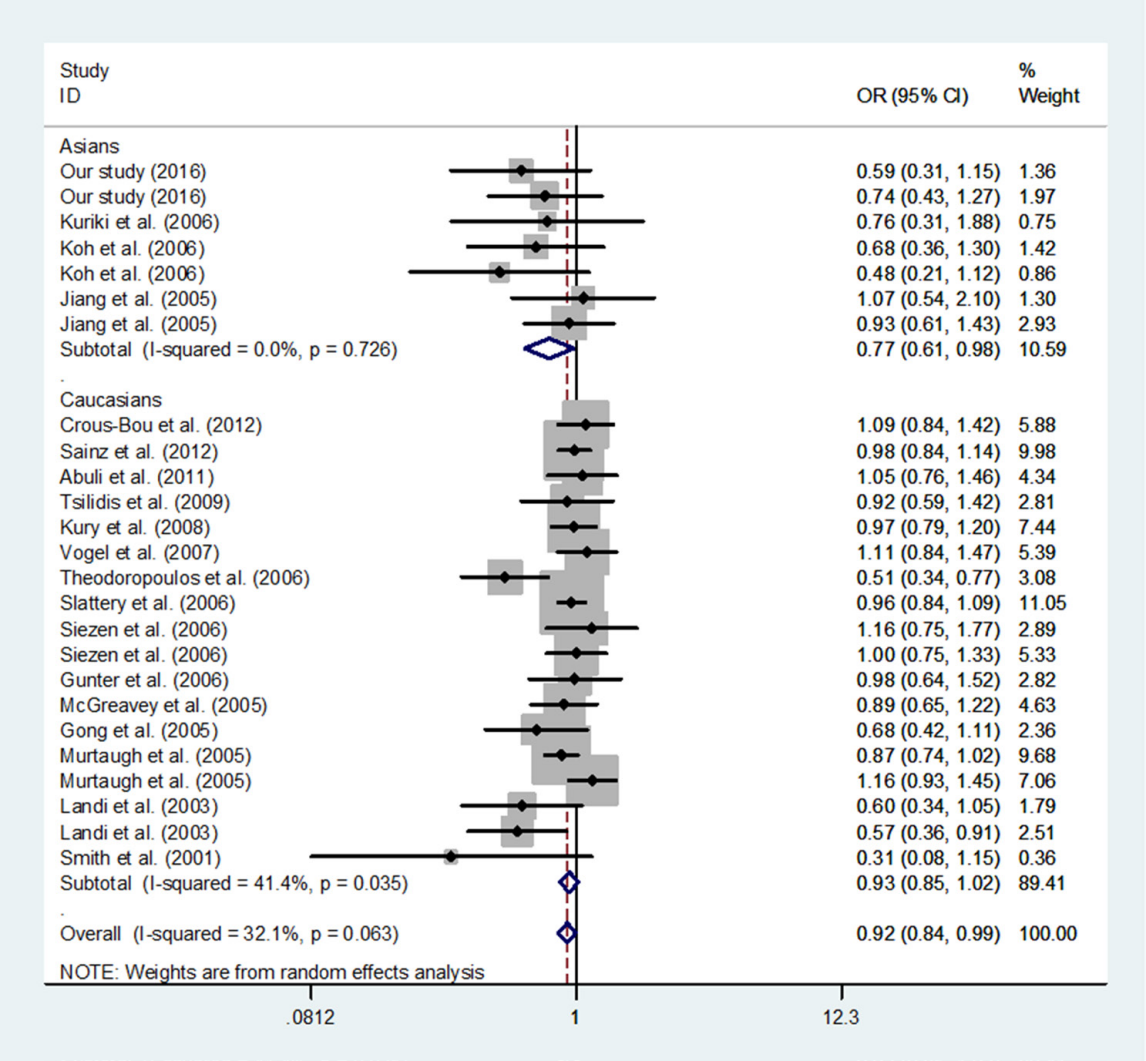
Forest plot of association between *PPARG* rs1801282 C>G polymorphism and CRC risk in random model (GG+CG vs. CC)

Overall, a significant association was identified between *PPARG* rs1801282 C>G polymorphism and decreased risk of CRC (G vs. C: OR = 0.94, 95% CI = 0.89–1.00, *P* = 0.040; GG+CG vs. CC: OR = 0.92, 95% CI = 0.84–0.99, *P* = 0.032, Figure 2). First, a further subgroup analysis was conducted by the ethnicity. Evidence of significant association between *PPARG* rs1801282 C>G polymorphism and decreased risk of CRC were also found among Asians (GG+CG vs. CC: OR = 0.76, 95% CI = 0.60–0.95, *P* = 0.018, Supplementary Table 1), but not Caucasians. Next, a further subgroup analysis was conducted by CRC region. *PPARG* rs1801282 C>G polymorphism was associated with decreased risk of colon cancer (G vs. C: OR = 0.66, 95% CI = 0.48–0.90, *P* = 0.009, GG+CG vs. CC: OR = 0.82, 95% CI = 0.71–0.94, *P* = 0.004, CG vs. CC + GG: OR = 0.70, 95% CI = 0.50–0.98, *P* = 0.035 and CG vs. CC: OR = 0.69, 95% CI = 0.49–0.96, *P* = 0.029; Supplementary Table 1), and rectum cancer (G vs. C: OR = 0.77, 95% CI = 0.59–0.99, *P* = 0.042, CG vs. CC + GG: OR = 0.73, 95% CI = 0.55–0.97, *P* = 0.032 and CG vs. CC: OR = 0.73, 95% CI = 0.55–0.97, *P* = 0.032; Supplementary Table 1), but not mixed type of CRC.

**Figure 3:**
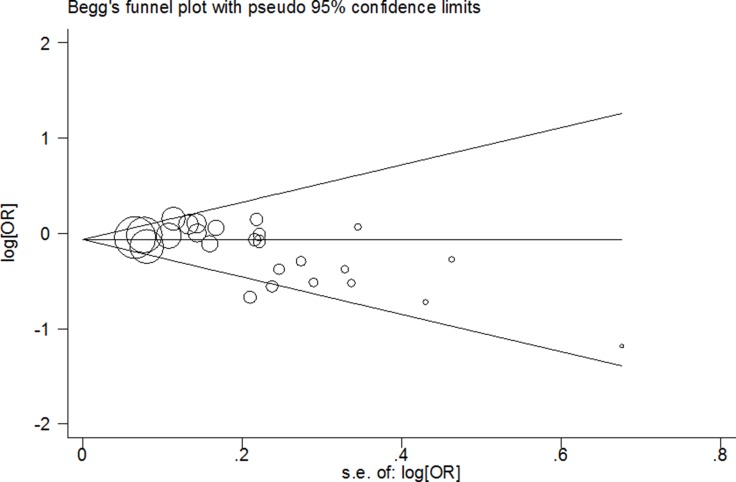
Begger’s funnel plot of the meta-analysis of between *PPARG* rs1801282 C>G polymorphism and CRC risk in random model (GG+CG vs. CC)

**Figure 4:**
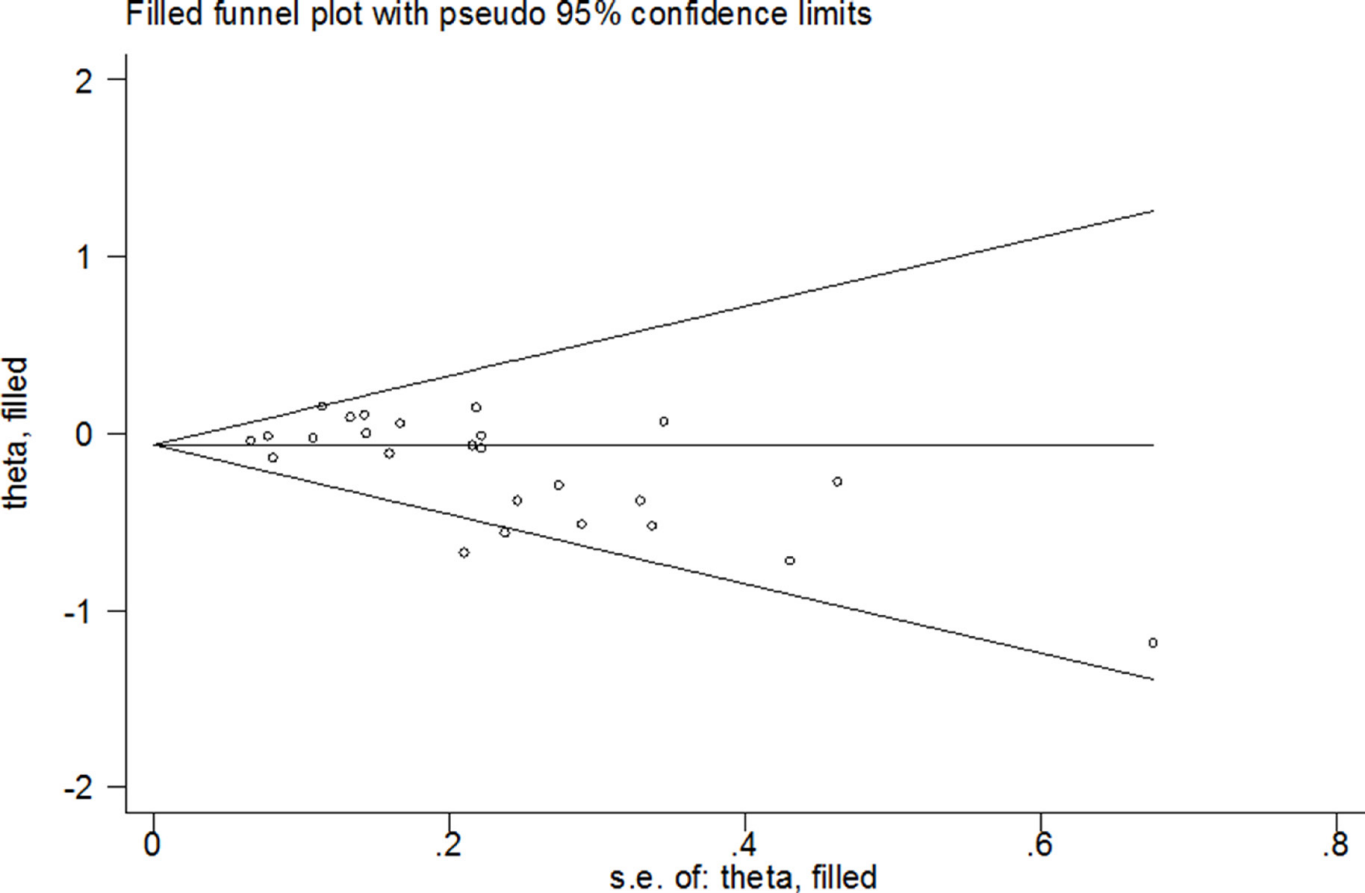
Filled funnel plot of the meta-analysis of between *PPARG* rs1801282 C>G polymorphism and CRC risk in random model (GG+CG vs. CC)

Both Begg’s test and Egger’s test were used to assess the potential publication bias in our study. It suggested that there was significant publication bias in some genetic models (G vs. C: Begg’s test *P* = 0.005, Egger’s test *P* = 0.009; GG vs. CC: Begg’s test *P* = 0.127, Egger’s test *P* = 0.026; GG+CG vs. CC: Begg’s test *P* = 0.005, Egger’s test *P* = 0.011; GG vs. CC+CG: Begg’s test *P* = 0.112, Egger’s test *P* = 0.024; CG vs. CC+GG: Begg’s test *P* = 0.010, Egger’s test *P* = 0.031 and CG vs. CC: Begg’s test *P* = 0.007, Egger’s test *P* = 0.026; Figure 3). Thus, adjusted ORs and CIs of nonparametric “trim-and-fill” method were harnessed to assess the stability of our findings. The adjusted ORs and CIs were: G vs. C: adjusted pooled OR = 0.94, 95% CI: 0.89–1.00, *P* = 0.054; GG vs. CC: adjusted pooled OR = 0.97, 95% CI: 0.76–1.23, *P* = 0.789; GG+CG vs. CC: adjusted pooled OR = 0.92, 95% CI: 0.85–0.99, *P* = 0.032; GG vs. CG+CC: adjusted pooled OR = 1.00, 95% CI: 0.79–1.26, *P* = 0.979; CG vs. CC+GG: adjusted pooled OR = 0.94, 95% CI: 0.88–1.01, *P* = 0.069 and CG vs. CC: adjusted pooled OR = 0.94, 95% CI: 0.88–1.00, *P* = 0.066 (Figure 4). These results suggested that our findings were stable.

**Figure 5:**
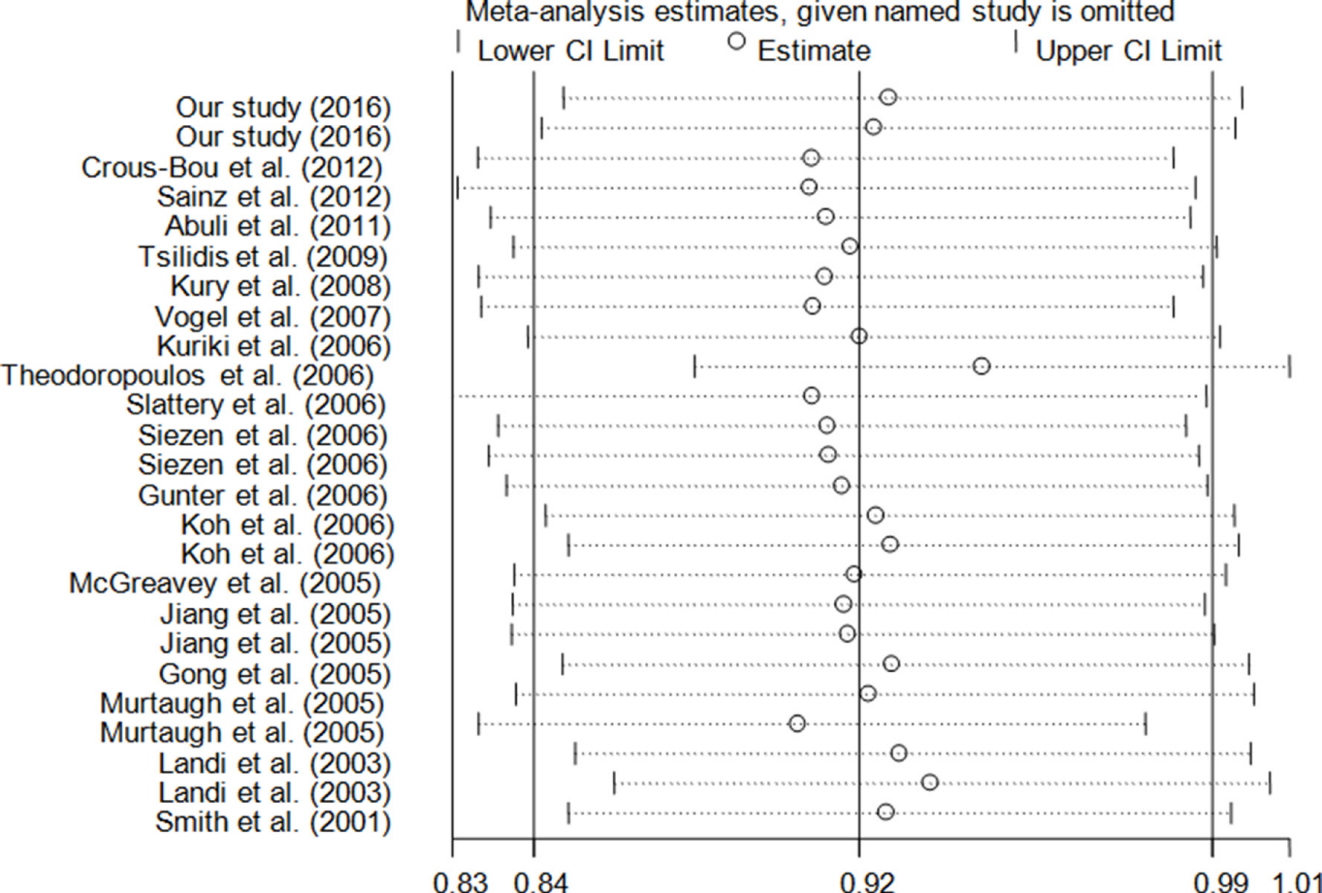
Sensitivity analysis on association between *PPARG* rs1801282 C>G polymorphism and CRC risk in random model (GG+CG vs. CC)

Using the exclusion method in turn, one-way sensitivity analysis was performed to determine whether an included study could affect the final decision. The results showed that our findings were stable and reliable (Figure 5).

For *PPARG* rs1801282 C>G polymorphism, the power value (α = 0.05) was 0.529 in G vs. C genetic model and 0.810 in GG/CG vs. CC genetic model among overall CRC cancer group, 0.717 in G vs. C genetic model, 0.791 in GG/CG vs. CC genetic model, 0.528 in CG vs. GG/CC genetic model and 0.562 in CG vs. CC genetic among colon cancer group, and 0.474 in G vs. C genetic model, 0.554 in CG vs. GG/CC genetic model and 0.552 in CG vs. CC genetic among rectum cancer group. In addition, for *PPARG* rs1801282 C>G, the power value was 0.660 in GG/CG vs. CC genetic model among Asians.

## DISCUSSION

PPARG is a nuclear hormone receptor, and mainly exists in colorectum, adipose tissue, and immune system [23]. PPARG plays a very important role in the inflammatory response, adipose cell differentiation, modulation of metabolism, and cellular apoptosis [24–27]. PPARG regulates and/or interacts with multifarious signaling pathways, including those associated with p21, p53, NF-kappa-β, STAT, BCL2, cyclooxygenase-2 (COX-2) and cyclin D1 [24–26, 28, 29]. PPARG is highly expressed in tumour cells, and treatment with PPARG ligands can induce cell apoptosis and differentiation [30–32]. *PPARG* mutation may increase CRC risk [22]. The possible association of *PPARG* rs1801282 C>G polymorphism with CRC risk has been extensively studied; however, findings of those investigations were conflicting, especially in Asians. To obtain a more precise assessment of these potential associations, we conducted a case-control study. Then, given the accumulating evidences and to shed some light on this issue, we performed a pooled-analysis of this potential relationship from Pubmed and EMBASE databases. For *PPARG* rs1801282 C>G polymorphism, individuals carrying the GG and GG/CG genotype had a tendency of decreased risk to CRC risk. In colon cancer subgroup, the results of logistic regression analyses indicated that tendency was also noted. The results of subsequent meta-analysis suggested that *PPARG* rs1801282 C>G polymorphism was associated with decreased susceptibility of CRC, especially in Asians, colon cancer and rectum cancer subgroups.

Adiposity and a sedentary lifestyle have been consistently related to CRC risk, and are vital determinants of hyperinsulinemia and insulin resistance. High concentrations of insulin or C-peptide (an insulin marker) have manifested direct association with CRC risk [33, 34]. A common functional polymorphism (Pro12Ala; rs1801282) in *PPARG* is C→G missense substitution causing a proline to alanine substitution in codon 12 of exon 2. Functional studies on *PPARG* rs1801282 polymorphism have revealed that G variant may alter the binding affinity of the protein to PPARG-responsive DNA elements compared to the C variant and the differential expression of PPARG-target genes has indicated the role of *PPARG* rs1801282 C>G polymorphism in transcriptional activity of PPARG [13, 35]. The *PPARG* rs1801282 C→G substitution produces protein with higher activity [13, 36]. Presence of the rs1801282 C>G polymorphism was reported to be associated with improved insulin sensitivity, lower body mass index (BMI), and a reduced risk of T2DM [37, 38]. Thus, it is possible that *PPARG* rs1801282 C>G polymorphism may be a protective factor for colorectal cancer through insulin-related mechanisms. In our case-control study and meta-analysis, we uniformly found that *PPARG* rs1801282 G allele might decrease CRC risk. These results were consistent with the protective effect of this polymorphism, and suggest this polymorphism may confer a lower CRC risk. Several meta-analyses have been undertaken to assess the relationship between *PPARG* rs1801282 C>G polymorphism and CRC risk [15, 16, 39]. In the present study, we also conducted a meta-analysis on this association including largest sample size (25 studies with 33,874 subjects). Overall, our findings of meta-analysis were consistent with those results. While in subgroup analyses, we found there were significant associations between *PPARG* rs1801282 C>G polymorphism and decreased risk of CRC among Asians, colon cancer and rectum cancer subgroups. These results of subgroup analysis were not similar to previous meta-analyses. In our meta-analysis, more studies and more participants were recruited. Thus, our findings may be more reliable than before.

Our study has some limitations. Firstly, our case-control study was hospital-based and might be unrepresentative of the Eastern Chinese Han population. Secondly, the sample size of patients with CRC was moderate. Thirdly, some factors, such as diet, physical activity, use of non-steroidal anti-inflammatory drugs, other functional SNPs in *PPARG* gene, and etc., were not considered. In the future, well-designed studies are needed to further investigate the association thoroughly. Finally, the relationship between *PPARG* polymorphisms and CRC risk involves a complex mechanism; thus, gene-gene and gene-environment interactions should be considered in future studies.

In conclusion, our study indicates that *PPARG* rs1801282 G allele might decrease the risk of overall CRC. In the future, more case-control studies with large sample size are needed to evaluate the effect of gene-gene and gene-environment interactions of the *PPARG* rs1801282 C>G with CRC risk.

## MATERIALS AND METHODS

### Study population and patient selection

Our study consisted of 387 CRC patients (236 men and 151 women) and 1,536 cancer-free controls (989 men and 547 women) in an Eastern Chinese Han population. The CRC cases were consecutively recruited from the Colorectal Surgery of Union Hospital, Fujian Medical University (Fuzhou city, China), between October 2014 and May 2016. Histologically, adenocarcinoma was confirmed via pathology. The major exclusion criteria were: patients with a history of another malignancy and hereditary nonpolyposis CRC. The controls were matched with age and gender and without any history of personal malignancy. All cancer-free controls were recruited from the Affiliated People’s Hospital of Jiangsu University and the Affiliated Union Hospital of Fujian Medical University. The variables and risk factors of all participants were collected by two doctors with a pre-structured questionnaire. All participants wrote the informed consent. Data on CRC clinicopathological characteristics were extracted from the medical records. This case-control study is approved by the ethics committee of Fujian Medical University and Jiangsu University (Fuzhou city and Zhenjiang city, China). The experimental protocol was performed in strict accordance with the approved guidelines.

### DNA extraction and genotyping

Every participant donated 2ml Ethylenediamine tetraacetic acid (EDTA)-anticoagulated intravenous blood. Genomic DNA from lymphocyte was extracted by Promega DNA Blood Mini Kit (Promega, Madison, USA). As described in previous studies, the genotyping of the rs1801282 C>G polymorphism in *PPARG* gene was performed by a custom-by-design 48-Plex SNPscan Kit (Genesky Biotechnologies Inc., Shanghai, China) [40, 41]. This genotyping method was based on double ligation and multiplex fluorescence PCR [42]. For quality control, 4% of all sample sizes (seventy-seven samples) were randomly selected and were genotyped again by the same genotyping method. The results of genotyping were not changed.

### Statistical analysis

Hardy-Weinberg equilibrium (HWE) was determined by an online Chi-square test (http://ihg.gsf.de/cgi-bin/hw/hwa1.pl). The association of *PPARG* rs1801282 C>G polymorphism with CRC risk was evaluated using crude and adjusted odds ratios (ORs) with their 95 % confidence intervals (CIs) when appropriate. All statistical analyses were performed by SAS 9.4 for Windows (SAS Institute, Cary, USA). An unpaired Student’s *t*-test was harnessed to check the differences for continuous variables between CRC cases and controls. And χ*2* test was used to assess the differences in the included risk factors [e.g., smoking, drinking and body mass index (BMI)], demographic variables, and the frequencies of various allele and genotype between CRC cases and controls. A *P* < 0.05 (two–tailed) was defined as the level of significance.

### Meta-analysis

To further assess the association of *PPARG* rs1801282 C>G polymorphism with CRC risk, we performed a comprehensive meta-analysis. Firstly, we carried out a systematic search through PubMed and EMBASE databases with the terms of ‘Peroxisome proliferator activated receptor gamma’ or ‘PPARG’ and ‘polymorphism’ or ‘mutation’ or ‘variant’ and ‘cancer’ or ‘carcinoma’ or ‘malignancy’ and ‘colorectal’ or ‘colon’ or ‘rectal’. All included publications were published up to 7 October 2016. The major included criteria were: (a) case–control or cohort study based on *PPARG* rs1801282 C>G polymorphism with sufficient genotype data and (b) the distribution of genotype in controls was in accord with HWE. The combined ORs and their 95% CIs were applied to determine the relationship of rs1801282 C>G polymorphism in *PPARG* gene with CRC risk. The between-study heterogeneity assumption was assessed using Chi-square-based statistic *I*^2^ test and Cochran’s Q-test [43]. When *I*^2^ > 50% or *P* < 0.1, we used the random-effects model (DerSimonian and Laird method) to estimate the pooled OR [44, 45]. Otherwise, the fixed effects model (the Mantel–Haenszel method) was applied [46]. Potential publication bias in meta-analysis was evaluated through Begg’s funnel plot and the Egger’s linear regression test [47] (*P* < 0.1 was defined representative of statistical publication bias). The statistical analyses of meta-analysis were performed by STATA version 12.0 (Stata Corporation, College Station, TX, USA). And all *P*-values were two-sided (*P* < 0.05). The power value of this meta-anlysis (α = 0.05) was evaluated by the Power and Sample Size Calculator (http://biostat.mc.vanderbilt.edu/twiki/bin/view/Main/PowerSampleSize) [48].

## SUPPLEMENTARY MATERIALS TABLE





## References

[1] Chen W, Zheng R, Baade PD, Zhang S, Zeng H, Bray F, Jemal A, Yu XQ, He J (2016). Cancer statistics in China, 2015. CA Cancer J Clin.

[2] Chen W, Zheng R, Zeng H, Zhang S, He J (2015). Annual report on status of cancer in China, 2011. Chin J Cancer Res.

[3] Weitz J, Koch M, Debus J, Hohler T, Galle PR, Buchler MW (2005). Colorectal cancer. Lancet.

[4] van Duijnhoven FJ, Bueno-De-Mesquita HB, Ferrari P, Jenab M, Boshuizen HC, Ros MM, Casagrande C, Tjonneland A, Olsen A, Overvad K, Thorlacius-Ussing O, Clavel-Chapelon F, Boutron-Ruault MC (2009). Fruit, vegetables, and colorectal cancer risk: the European Prospective Investigation into Cancer and Nutrition. Am J Clin Nutr.

[5] Gerber M (2009). Background review paper on total fat, fatty acid intake and cancers. Ann Nutr Metab.

[6] Xu M, Chen YM, Huang J, Fang YJ, Huang WQ, Yan B, Lu MS, Pan ZZ, Zhang CX (2016). Flavonoid intake from vegetables and fruits is inversely associated with colorectal cancer risk: a case-control study in China. Br J Nutr.

[7] Nagle CM, Wilson LF, Hughes MC, Ibiebele TI, Miura K, Bain CJ, Whiteman DC, Webb PM (2015). Cancers in Australia in 2010 attributable to inadequate consumption of fruit, non-starchy vegetables and dietary fibre. Aust N Z J Public Health.

[8] Weigl K, Jansen L, Chang-Claude J, Knebel P, Hoffmeister M, Brenner H (2016). Family history and the risk of colorectal cancer: The importance of patients’ history of colonoscopy. Int J Cancer.

[9] Lowery JT, Ahnen DJ, Schroy PC, Hampel H, Baxter N, Boland CR, Burt RW, Butterly L, Doerr M, Doroshenk M, Feero WG, Henrikson N, Ladabaum U (2016). Understanding the contribution of family history to colorectal cancer risk and its clinical implications: A state-of-the-science review. Cancer.

[10] Guazzoni G, Montorsi F, Colombo R, Di Girolamo V, Da Pozzo L, Rigatti P (1991). Long term experience with the prostatic spiral for urinary retention due to benign prostatic hyperplasia. Scand J Urol Nephrol.

[11] AlSaleh A, Sanders TA, O’Dell SD (2012). Effect of interaction between PPARG, PPARA and ADIPOQ gene variants and dietary fatty acids on plasma lipid profile and adiponectin concentration in a large intervention study. Proc Nutr Soc.

[12] Barbieri M, Rizzo MR, Papa M, Acampora R, De Angelis L, Olivieri F, Marchegiani F, Franceschi C, Paolisso G (2005). Role of interaction between variants in the PPARG and interleukin-6 genes on obesity related metabolic risk factors. Exp Gerontol.

[13] Deeb SS, Fajas L, Nemoto M, Pihlajamaki J, Mykkanen L, Kuusisto J, Laakso M, Fujimoto W, Auwerx J (1998). A Pro12Ala substitution in PPARgamma2 associated with decreased receptor activity, lower body mass index and improved insulin sensitivity. Nat Genet.

[14] Du J, Shi H, Lu Y, Du W, Cao Y, Li Q, Ma J, Ye X, Cheng J, Yu X, Gao Y, Zhou L (2011). Tagging single nucleotide polymorphisms in the PPAR-gamma and RXR-alpha gene and type 2 diabetes risk: a case-control study of a Chinese Han population. J Biomed Res.

[15] Wang W, Shao Y, Tang S, Cheng X, Lian H, Qin C (2015). Peroxisome proliferator-activated receptor-gamma (PPARgamma) Pro12Ala polymorphism and colorectal cancer (CRC) risk. Int J Clin Exp Med.

[16] Wei Z, Han G, Bai X (2015). Effect of Proliferator-Activated Receptor-gamma Pro12Ala Polymorphism on Colorectal Cancer Risk: A Meta-Analysis. Med Sci Monit.

[17] Jiang J, Gajalakshmi V, Wang J, Kuriki K, Suzuki S, Nakamura S, Akasaka S, Ishikawa H, Tokudome S (2005). Influence of the C161T but not Pro12Ala polymorphism in the peroxisome proliferator-activated receptor-gamma on colorectal cancer in an Indian population. Cancer Sci.

[18] Kuriki K, Hirose K, Matsuo K, Wakai K, Ito H, Kanemitsu Y, Hirai T, Kato T, Hamajima N, Takezaki T, Suzuki T, Saito T, Tanaka R (2006). Meat, milk, saturated fatty acids, the Pro12Ala and C161T polymorphisms of the PPARgamma gene and colorectal cancer risk in Japanese. Cancer Sci.

[19] Koh WP, Yuan JM, Van Den Berg D, Ingles SA, Yu MC (2006). Peroxisome proliferator-activated receptor (PPAR) gamma gene polymorphisms and colorectal cancer risk among Chinese in Singapore. Carcinogenesis.

[20] Siezen CL, Bueno-de-Mesquita HB, Peeters PH, Kram NR, van Doeselaar M, van Kranen HJ (2006). Polymorphisms in the genes involved in the arachidonic acid-pathway, fish consumption and the risk of colorectal cancer. Int J Cancer.

[21] Landi S, Moreno V, Gioia-Patricola L, Guino E, Navarro M, de Oca J, Capella G, Canzian F, Bellvitge Colorectal Cancer Study G (2003). Association of common polymorphisms in inflammatory genes interleukin (IL)6, IL8, tumor necrosis factor alpha, NFKB1, and peroxisome proliferator-activated receptor gamma with colorectal cancer. Cancer Res.

[22] Murtaugh MA, Ma KN, Caan BJ, Sweeney C, Wolff R, Samowitz WS, Potter JD, Slattery ML (2005). Interactions of peroxisome proliferator-activated receptor {gamma} and diet in etiology of colorectal cancer. Cancer Epidemiol Biomarkers Prev.

[23] Tontonoz P, Hu E, Spiegelman BM (1994). Stimulation of adipogenesis in fibroblasts by PPAR gamma 2, a lipid-activated transcription factor. Cell.

[24] Elrod HA, Sun SY (2008). PPARgamma and Apoptosis in Cancer. PPAR Res.

[25] Girnun GD, Smith WM, Drori S, Sarraf P, Mueller E, Eng C, Nambiar P, Rosenberg DW, Bronson RT, Edelmann W, Kucherlapati R, Gonzalez FJ, Spiegelman BM (2002). APC-dependent suppression of colon carcinogenesis by PPARgamma. Proc Natl Acad Sci USA.

[26] Sarraf P, Mueller E, Jones D, King FJ, DeAngelo DJ, Partridge JB, Holden SA, Chen LB, Singer S, Fletcher C, Spiegelman BM (1998). Differentiation and reversal of malignant changes in colon cancer through PPARgamma. Nat Med.

[27] Tontonoz P, Spiegelman BM (2008). Fat and beyond: the diverse biology of PPARgamma. Annu Rev Biochem.

[28] Subbaramaiah K, Lin DT, Hart JC, Dannenberg AJ (2001). Peroxisome proliferator-activated receptor gamma ligands suppress the transcriptional activation of cyclooxygenase-2. Evidence for involvement of activator protein-1 and CREB-binding protein/p300. J Biol Chem.

[29] Yang WL, Frucht H (2001). Activation of the PPAR pathway induces apoptosis and COX-2 inhibition in HT-29 human colon cancer cells. Carcinogenesis.

[30] Wan Z, Shi W, Shao B, Shi J, Shen A, Ma Y, Chen J, Lan Q (2011). Peroxisome proliferator-activated receptor gamma agonist pioglitazone inhibits beta-catenin-mediated glioma cell growth and invasion. Mol Cell Biochem.

[31] Mannelli M, Cantini G, Poli G, Mangoni M, Nesi G, Canu L, Rapizzi E, Borgogni E, Ercolino T, Piccini V, Luconi M (2010). Role of the PPAR-gamma system in normal and tumoral pituitary corticotropic cells and adrenal cells. Neuroendocrinology.

[32] Schmidt MV, Brune B, von Knethen A (2010). The nuclear hormone receptor PPARgamma as a therapeutic target in major diseases. Scientific World Journal.

[33] Jenab M, Riboli E, Cleveland RJ, Norat T, Rinaldi S, Nieters A, Biessy C, Tjonneland A, Olsen A, Overvad K, Gronbaek H, Clavel-Chapelon F, Boutron-Ruault MC (2007). Serum C-peptide, IGFBP-1 and IGFBP-2 and risk of colon and rectal cancers in the European Prospective Investigation into Cancer and Nutrition. Int J Cancer.

[34] Wei EK, Ma J, Pollak MN, Rifai N, Fuchs CS, Hankinson SE, Giovannucci E (2005). A prospective study of C-peptide, insulin-like growth factor-I, insulin-like growth factor binding protein-1, and the risk of colorectal cancer in women. Cancer Epidemiol Biomarkers Prev.

[35] Masugi J, Tamori Y, Mori H, Koike T, Kasuga M (2000). Inhibitory effect of a proline-to-alanine substitution at codon 12 of peroxisome proliferator-activated receptor-gamma 2 on thiazolidinedione-induced adipogenesis. Biochem Biophys Res Commun.

[36] Yen CJ, Beamer BA, Negri C, Silver K, Brown KA, Yarnall DP, Burns DK, Roth J, Shuldiner AR (1997). Molecular scanning of the human peroxisome proliferator activated receptor gamma (hPPAR gamma) gene in diabetic Caucasians: identification of a Pro12Ala PPAR gamma 2 missense mutation. Biochem Biophys Res Commun.

[37] Douglas JA, Erdos MR, Watanabe RM, Braun A, Johnston CL, Oeth P, Mohlke KL, Valle TT, Ehnholm C, Buchanan TA, Bergman RN, Collins FS, Boehnke M (2001). The peroxisome proliferator-activated receptor-gamma2 Pro12A1a variant: association with type 2 diabetes and trait differences. Diabetes.

[38] Mori H, Ikegami H, Kawaguchi Y, Seino S, Yokoi N, Takeda J, Inoue I, Seino Y, Yasuda K, Hanafusa T, Yamagata K, Awata T, Kadowaki T (2001). The Pro12 –>Ala substitution in PPAR-gamma is associated with resistance to development of diabetes in the general population: possible involvement in impairment of insulin secretion in individuals with type 2 diabetes. Diabetes.

[39] Chen C, Wang L, Liao Q, Xu L, Huang Y, Zhang C, Ye H, Xu X, Ye M, Duan S (2014). Association between six genetic polymorphisms and colorectal cancer: a meta-analysis. Genet Test Mol Biomarkers.

[40] Zheng L, Yin J, Wang L, Wang X, Shi Y, Shao A, Tang W, Ding G, Liu C, Chen S, Gu H (2013). Interleukin 1B rs16944 G>A polymorphism was associated with a decreased risk of esophageal cancer in a Chinese population. Clin Biochem.

[41] Yin J, Wang L, Shi Y, Shao A, Tang W, Wang X, Ding G, Liu C, Chen S, Gu H (2014). Interleukin 17A rs4711998 A>G polymorphism was associated with a decreased risk of esophageal cancer in a Chinese population. Dis Esophagus.

[42] Yin J, Wang X, Wei J, Wang L, Shi Y, Zheng L, Tang W, Ding G, Liu C, Liu R, Chen S, Xu Z, Gu H (2015). Interleukin 12B rs3212227 T > G polymorphism was associated with an increased risk of gastric cardiac adenocarcinoma in a Chinese population. Dis Esophagus.

[43] Higgins JP, Thompson SG (2002). Quantifying heterogeneity in a meta-analysis. Stat Med.

[44] Higgins JP, Thompson SG, Deeks JJ, Altman DG (2003). Measuring inconsistency in meta-analyses. BMJ.

[45] DerSimonian R, Laird N (1986). Meta-analysis in clinical trials. Control Clin Trials.

[46] Mantel N, Hanzel W (1959). Statistical aspects of the analysis of data from retrospective studies of disease. J Natl Cancer Inst.

[47] Egger M, Davey Smith G, Schneider M, Minder C (1997). Bias in meta-analysis detected by a simple, graphical test. BMJ.

[48] Tang W, Qiu H, Ding H, Sun B, Wang L, Yin J, Gu H (2013). Association between the STK15 F31I polymorphism and cancer susceptibility: a meta-analysis involving 43,626 subjects. PLoS One.

