# Supplementary material for: Association of PPARG rs 1801282 C>G polymorphism with risk of colorectal cancer: from a case-control study to a meta-analysis
Source: Oncotarget. 2017 Aug 10;8(59):100558–69. doi: 10.18632/oncotarget.20138 (PMC5725043; doi:10.18632/oncotarget.20138)
Supplement: Supplementary file 1 [file oncotarget-08-100558-s001.pdf]

Association of *PPARG* rs 1801282 C>G polymorphism with risk of colorectal cancer: from a case-control study to a meta-analysis

SUPPLEMENTARY MATERIALS

Supplementary Table 1: Meta-analysis of the *PPARG* rs1801282 C>G polymorphism and CRC risk.
See_Supplementary_ Table 1
