# Supplementary material for: Association of PPARG rs 1801282 C>G polymorphism with risk of colorectal cancer: from a case-control study to a meta-analysis
Source: Oncotarget. 2017 Aug 10;8(59):100558–69. doi: 10.18632/oncotarget.20138 (PMC5725043; doi:10.18632/oncotarget.20138)
Supplement: Supplementary file 2 [file oncotarget-08-100558-s002.docx]

**Supplementary Table 1:** Meta-analysis of the *PPARG* rs1801282 C>G polymorphism and CRC risk

|  | No. of study | G vs. C | | |  | GG vs. CC | | |  | GG+CG vs. CC | | |  | GG vs. CC+CG | | |  | CG vs. CC+GG | | |  | CG vs. CC | | |
| --- | --- | --- | --- | --- | --- | --- | --- | --- | --- | --- | --- | --- | --- | --- | --- | --- | --- | --- | --- | --- | --- | --- | --- | --- |
|  |  | OR(95%CI) | *P* | *P*het |  | OR(95%CI) | *P* | *P*het |  | OR(95%CI) | *P* | *P*het |  | OR(95%CI) | *P* | *P*het |  | OR(95%CI) | *P* | *P*het |  | OR(95%CI) | *P* | *P*het |
| Overall | 25 | **0.94(0.89-1.00)** | **0.040** | 0.144 |  | 0.92(0.73-1.15) | 0.459 | 0.818 |  | **0.92(0.84-0.99)** | **0.032** | 0.063 |  | 0.94(0.75-1.19) | 0.611 | 0.863 |  | 0.94(0.88-1.00) | 0.053 | 0.165 |  | 0.94(0.88-1.00) | 0.050 | 0.133 |
| Type |  |  |  |  |  |  |  |  |  |  |  |  |  |  |  |  |  |  |  |  |  |  |  |  |
| Colon cancer | 5 | **0.66(0.48-0.90)** | **0.009** | 0.340 |  | 0.43(0.10-1.79) | 0.243 | 0.282 |  | **0.82(0.71-0.94)** | **0.004** | 0.324 |  | 0.44(0.10-1.86) | 0.265 | 0.288 |  | **0.70(0.50-0.98)** | **0.035** | 0.249 |  | **0.69(0.49-0.96)** | **0.029** | 0.257 |
| Rectum cancer | 6 | **0.77(0.59-0.99)** | **0.042** | 0.277 |  | 0.96(0.40-2.32) | 0.925 | 0.794 |  | 0.78(0.56-1.09) | 0.140 | 0.085 |  | 1.02(0.42-2.46) | 0.969 | 0.802 |  | **0.73(0.55-0.97)** | **0.032** | 0.394 |  | **0.73(0.55-0.97)** | **0.032** | 0.380 |
| Mixed type | 14 | 0.97(0.91-1.03) | 0.284 | 0.442 |  | 0.94(0.74-1.20) | 0.622 | 0.646 |  | 0.96(0.90-1.03) | 0.277 | 0.325 |  | 0.96(0.76-1.23) | 0.765 | 0.716 |  | 0.96(0.90-1.03) | 0.307 | 0.346 |  | 0.96(0.90-1.03) | 0.305 | 0.298 |
| Ethnicity |  |  |  |  |  |  |  |  |  |  |  |  |  |  |  |  |  |  |  |  |  |  |  |  |
| Asians | 7 | 0.83(0.65-1.06) | 0.128 | 0.787 |  | 1.03(0.38-2.82) | 0.903 | 0.905 |  | **0.76(0.60-0.95)** | **0.018** | 0.726 |  | 1.05(0.39-2.85) | 0.925 | 0.894 |  | 0.82(0.63-1.06) | 0.129 | 0.628 |  | 0.81(0.63-1.06) | 0.127 | 0.644 |
| Caucasians | 18 | 0.93(0.85-1.01) | 0.094 | 0.065 |  | 0.91(0.72-1.15) | 0.438 | 0.619 |  | 0.93(0.85-1.02) | 0.123 | 0.035 |  | 0.94(0.74-1.19) | 0.586 | 0.690 |  | 0.93(0.85-1.03) | 0.151 | 0.094 |  | 0.93(0.84-1.02) | 0.140 | 0.071 |

*P*het: *P* value for heterogeneity

Bold values are statistically significant (*P* <0.05)
